# Hospital healthcare utilisation in patients with atrial fibrillation: the role of multimorbidity and age

**DOI:** 10.1007/s12471-025-01968-x

**Published:** 2025-07-18

**Authors:** Melissa E. Middeldorp, Colinda van Deutekom, Liann I. Weil, Ursula W. De Ruijter, Patrick T. Jeurissen, Isabelle C. Van Gelder, Barbara C. van Munster, Michiel Rienstra

**Affiliations:** 1 Department of Cardiology, University of Groningen, University Medical Centre Groningen, Groningen, The Netherlands; 2 Department of Geriatric Medicine, University of Groningen, University Medical Centre Groningen, Groningen, The Netherlands; 3 Department of Public Health, Erasmus University Medical Centre, Rotterdam, The Netherlands; 4 Radboud Institute for Health Sciences, Scientific Center for Quality of Healthcare (IQ healthcare), Radboud University Medical Centre, Nijmegen, The Netherlands

**Keywords:** Atrial fibrillation, Healthcare utilisation, Multimorbidity, Comorbidities, Age

## Abstract

**Background:**

Patients with atrial fibrillation (AF) often present with multimorbidity and may require a higher healthcare utilisation. We aimed to compare hospital healthcare utilisation among AF patients to non-cardiovascular disease (non-CVD) patients and explore the role of multimorbidity and age.

**Methods:**

We performed a retrospective cohort study using electronic health records data from three hospitals in the Netherlands. Patients aged ≥ 18 years with ≥ 1 inpatient or outpatient presentation were included. Diagnoses were determined using the International Classification of Diseases and Related Health Problems 10 codes and linked with the Dutch Hospital Data Clinical Classification Software to determine comorbidities.

**Results:**

A total of 226,991 patients, 5,127 (2%) had AF. AF patients had significantly more outpatient visits (6.6 vs 3.6), emergency department visits (0.9 vs 0.2), and in-hospital days (4.0 vs 1.5) compared to non-CVD patients/year (all *p* < 0.001). AF patients saw more frequently multiple specialists, (13% vs 2% consulting ≥ 5 specialists, *p* < 0.001). Number of outpatient visits for AF patients increased with number of comorbidities: from a median of 1 (0–1 comorbidities) to 11 (≥ 4 comorbidities) (*p* < 0.001). Similarly, in-hospital days increased from 0.6 days (0–1 comorbidities) to 8.2 days (≥ 4 comorbidities) (*p* < 0.001). Regardless of age, AF patients had more outpatient and emergency department visits and more days in hospital days compared to non-CVD patients (all *p* < 0.001).

**Conclusions:**

Patients with AF had significantly greater hospital healthcare utilisation use compared to non-CVD patients, independent of age. Therefore, there is a need for more cohesive care pathways in AF patients to reduce healthcare utilisation.

**Supplementary Information:**

The online version of this article (10.1007/s12471-025-01968-x) contains supplementary material, which is available to authorized users.

## What’s new

- Compared to patients without cardiovascular disease patients with AF had significantly more hospital healthcare utilisation.
- There was a higher number of outpatient and emergency visits as well as in hospital days associated with an increase with comorbidities and age.
- This study highlights the need for improved management of multimorbidity in patients with atrial fibrillation. Implementation of better care pathways aligning specialist care could help to reduce the burden of atrial fibrillation on healthcare systems

## Introduction

Atrial fibrillation (AF) is the most prevalent sustained cardiac arrhythmia [1]. Hospitalisations due to AF continue to increase [2] due to lifestyle changes, multimorbidity, and an ageing population now surpassing myocardial infarction and heart failure hospitalisation, leading to high rates of healthcare system utilisation [3]. The cause of hospitalisation among patients with AF differs based on age. A large cohort study showed that 42% of those aged hospitalisations < 65 years were due to AF, whereas aged > 75 years 49% of hospitalisations were due to other cardiovascular diseases (CVD) [4].

AF is a complex disease associated with comorbidities and risk factors, including ageing, obesity, hypertension, heart failure, diabetes mellitus, and vascular disease all of which contribute to worse AF outcomes [5, 6]. AF often coexists with multiple comorbidities, multimorbidity, defined as two or more comorbidities [7]. Data from population cohorts reports multimorbidity present in approximately 80% of patients with incident AF [8]. Patients with AF and four or more comorbid conditions showed a six-fold increased risk of all-cause mortality [8].

**Fig. 1:**
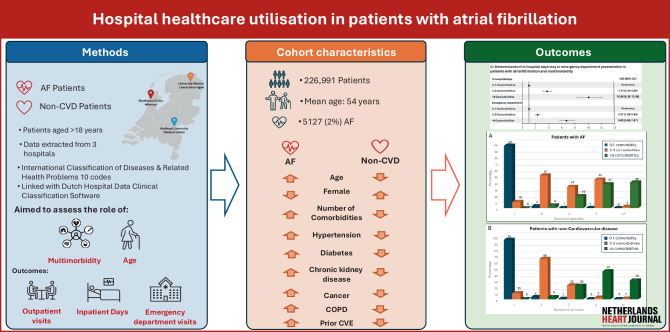
Infographic: Hospital healthcare utilisation in patients with atrial fibrillation. Overview of the study cohort with recruitment from 3 centres in the Netherlands, aims and outcomes. Methodology including baseline characteristics and key findings from the study. (*AF* atrial fibrillation, *CVD* cardiovascular disease, *COPD* chronic obstructive pulmonary disease, *CVE* cerebrovascular event)

A notable gap persists in understanding the impact of AF-related multimorbidity and AF itself on healthcare utilisation. Our aim was to investigate hospital healthcare utilisation among AF patients compared to non-CVD patients who presented to a hospital for at least one inpatient or outpatient service in the Netherlands. Additionally, we explored the role of multimorbidity and age in healthcare utilisation (Fig. 1).

## Methods

We performed a retrospective cohort analysis using administrative electronic health record data from one general hospital and two academic medical centres in the Netherlands. The general hospital was Northwest Clinics Alkmaar (NWCA), and the two academic centres were the University Medical Centre Groningen (UMCG) and Radboud University Medical Centre (Radboud UMC).

### Data extraction

We included 252,623 patients between 2017–2019. For the primary comparison, we excluded patients with CVD but without AF (*n* = 25,643), resulting in a final analytic sample of patients with AF and comorbid CVD, and those with neither AF nor CVD. This allowed us to establish a clean comparator group without CVD. Hospital data and healthcare utilisation were collected based on the coding for hospital care and claiming payments commonly used in the Netherlands, known as Diagnosis-Treatment Combinations (DTCs) [9]. Data were collected from each hospital, including age, sex, in-hospital days, emergency department, and outpatient visits. Additionally, we collected the type and number of diagnoses for each outpatient visit and the number and type of medical specialists involved per outpatient visit per patient. All patients aged 18 years or older who presented to the hospital were included in the analysis. Patient data were de-identified, and patients who declined the use of their data were excluded prior to data collection.

The Central Ethics Review Board of the UMCG approved the pseudonymous use of data for research purposes and granted a waiver of informed consent for all three hospitals. This approval was recognised by the Radboud UMC and NWCA for the use of their data. Data transfer agreements were signed between the UMCG, the Radboud UMC, and NWCA.

### Diagnosis codes and definitions

To determine the diagnosis of patients, diagnoses within the Diagnosis-Treatment Combinations were used and registered with the International Classification of Diseases and Related Health Problems, 10th Revision (ICD-10) codes, and linked to the Dutch Hospital Data–Clinical Classification Software (DHD-CCS) [10]. To define patients with AF, we used the DHD-CCS coding for atrial arrhythmias. To determine multimorbidity, the number of comorbidities were defined based on the number of diagnoses deemed by the DHD-CCS classification code. Non-CVD patients included those patients not having a diagnosis of acute or old myocardial infarction, congestive heart failure, valvular disease, coronary atherosclerosis, cardiac congenital anomalies, or any thromboembolism.

### Statistical analysis

Baseline characteristics are reported for patients with AF and non-CVD patients. Variables are presented as means (standard deviation (SD)) for continuous variables when normally distributed, and as median (quintiles) when not normally distributed. For categorical variables we reported frequencies (percentages). The groups were compared using *t*-test or Kruskal-Wallis tests (if the normality assumption was violated) for continuous variables and χ^2^ or Fisher’s Exact test for categorical variables. Pearson’s correlation coefficients were calculated to assess the relationship between healthcare utilisation and diagnostic categories for AF and non-CVD patients. To compare the correlations between groups, Fisher’s *r*-to-*z* transformation was applied, converting correlation coefficients to *z*-scores for comparison. The *z*-statistic was calculated as the difference between the transformed correlations, divided by the standard error. To determine the impact of multimorbidity on healthcare utilisation, a stratified analysis was conducted in 3 categories: 0–1 comorbidities, 2–3 comorbidities, or ≥ 4 comorbidities. An additional age-stratified analysis was performed in 3 categories: 18–59 years, 60–74 years and ≥ 75 years to determine the impact of age. Logistic regression analysis was done to determine the association between individual comorbidities and healthcare utilisation. For patients with AF, regression models were used to determine the association between AF and healthcare utilisation, both unadjusted and adjusted for age and sex. Data processing was performed using R (Version 4.3.3). Statistical analyses were performed with STATA software (version 18.0). A *p*-value < 0.05 was considered statistically significant.

## Results

We included a total of 226,991 patients with a mean age of 53.7 ± 18.1 of whom 5,127 (2%) had AF: 59,334 from UMCG (1,448, 28%), 141,753 from Radboud UMC (2,152, 42%), and 25,904 from NWCA (1,527, 30%). Patients with AF were older and presented with significantly more comorbidities compared to those without CVD (Table 1).

**Table 1:** Patient characteristics and hospital healthcare utilisation in patients with AF compared to those with non-CVD

	AF	Non-CVD	Total	*p*-value
	5,127	221,864	226,991	
Age (SD)	67.9 ± 12.3	53.4 ± 18.1	53.7 ± 18.1	< 0.001
Female, *n* (%)	2,274 (44)	123,148 (56)	126,422 (56)	< 0.001
Number of comorbidities, mean (SD)	2.9 ± 1.9	1.8 ± 1.1	1.7 ± 1.2	< 0.001
Hypertension, *n* (%)	84 (2)	2,159 (0.9)	2,243 (0.9)	< 0.001
Heart Failure, *n* (%)	222 (4)	0	222 (0.1)	< 0.001
Diabetes, *n* (%)	118 (2.3)	3,703 (2)	3,821 (2)	0.001
Myocardial infarction, *n* (%)	38 (1)	0	38 (0.1)	< 0.001
Valvular disease, *n* (%)	63 (1)	0	63 (0.1)	< 0.001
Syncope, *n* (%)	26 (0.5)	532 (0.2)	558 (0.3)	< 0.001
Chronic kidney disease, *n* (%)	154 (3)	2,091 (1)	2,245 (1)	< 0.001
Cancer, *n* (%)	1,203 (24)	45,209 (20)	46,412 (21)	< 0.001
Prior cerebrovascular event*, *n* (%)	100 (2)	2,061 (0.9)	2,161 (1)	< 0.001
COPD, *n* (%)	183 (4)	3,026 (1)	3,209 (1)	< 0.001
*Hospital healthcare utilisation*				
Outpatient visits (SD)	6.6 ± 6.2	3.6 ± 4.1	3.6 ± 1.2	< 0.001
Outpatient visits, median (Q1, Q3)	5 (2, 9)	2 (1, 4)	2 (1, 4)	< 0.001
ED Visits (SD)	0.9 ± 1.4	0.2 ± 0.7	0.2 ± 0.7	< 0.001
ED visits, median (Q1, Q3)	0 (0, 1)	0 (0, 0)	0 (0, 0)	< 0.001
In hospital days (SD)	4.0 ± 8.6	1.5 ± 6.2	1.6 ± 6.2	< 0.001
In hospital days, median (Q1, Q3)	0 (0, 4)	0 (0, 0)	0 (0, 0)	< 0.001
*Involved medical specialties*, *n* (%)				
1	1,507 (29)	134,970 (61)	136,477 (60)	
2	1,214 (24)	52,869 (24)	54,083 (24)	
3	1,032 (20)	20,388 (9)	21,420 (9)	< 0.001
4	692 (14)	8,399 (4)	9,091 (4)	
5+	682 (13)	5,238 (2)	5,920 (3)	
Involved specialties, mean (SD)	2.7 ± 1.6	1.6 ± 1.03	1.7 ± 1.06	< 0.001
*Medical specialist*				
Anaesthesiology, *n* (%)	121 (2)	5,788 (3)	5,909 (3)	< 0.001
Cardiology, *n* (%)	4,427 (86)	14,436 (7)	18,863 (8)	< 0.001
Cardiothoracic surgery, *n* (%)	87 (2)	496 (0.2)	583 (0.2)	< 0.001
Clinical genetics, *n* (%)	41 (0.8)	12,060 (5)	12,101 (6)	< 0.001
Dermatology, *n* (%)	502 (10)	21,942 (10)	22,444 (10)	0.815
Gastroenterology, *n* (%)	506 (10)	13,318 (6)	13,824 (6)	< 0.001
General surgery, *n* (%)	796 (16)	26,725 (12)	27,521 (12)	< 0.001
Geriatrics, *n* (%)	198 (4)	3,050 (1)	3,248 (1)	< 0.001
Gynaecology, *n* (%)	221 (4)	27,043 (12)	27,264 (12)	< 0.001
Internal medicine, *n* (%)	1,477 (29)	47,174 (21)	48,651 (21)	< 0.001
Neurology, *n* (%)	831 (16)	27,854 (13)	28,685 (13)	< 0.001
Neurosurgery, *n* (%)	89 (2)	6,323 (3)	6,412 (3)	< 0.001
Ophthalmology, *n* (%)	462 (9)	24,376 (11)	24,838 (11)	< 0.001
Orthopaedic surgery, *n* (%)	432 (8)	17,712 (8)	18,144 (8)	0.248
Otorhinolaryngology, *n* (%)	382 (8)	18,874 (9)	19,256 (9)	0.007
Physiatry Rehabilitation, *n* (%)	41 (1)	2,965 (1)	3,006 (1)	0.001
Plastic surgery, *n* (%)	105 (2)	8,285 (4)	8,390 (4)	< 0.001
Psychiatry, *n* (%)	7 (0.1)	1,586 (1)	1,592 (1)	< 0.001
Pulmonology, *n* (%)	740 (14)	13,819 (6)	14,559 (6)	< 0.001
Radiotherapy, *n* (%)	463 (9)	13,058 (6)	13,521 (6)	< 0.001
Rheumatology, *n* (%)	328 (6)	11,158 (5)	11,486 (5)	< 0.001
Urology, *n* (%)	475 (9)	16,383 (7)	16,858 (7)	< 0.001

*AF* atrial fibrillation, *COPD* chronic obstructive pulmonary disease, *CVD* cardiovascular disease, *ED* emergency department

* Cerebrovascular event was defined as intracerebral hematoma, chronic subdural hematoma/hygroma, cerebrovascular accident/Transient ischemic attack, or, intracranial, subarachnoidal or intracerebral hemorrhage

### Healthcare utilisation in AF versus non-CVD patients

Patients diagnosed with AF had a significantly more outpatient visits, emergency department visits, and in-hospital days compared to non-CVD patients (Table 1). AF patients had more outpatient visits with ≥ 5 medical specialists compared to non-CVD patients (13% vs 2%, *p* < 0.001). The mean number of specialists involved in care of AF patients was 2.7 ± 1.6 compared to 1.6 ± 1.0 in non-CVD patients (*p* < 0.001). The type of medical specialists consulted by patients are shown in Table 1.

### Multimorbidity in healthcare utilisation in AF versus non-CVD patients

In AF patients, the mean age increased with a number of comorbidities, ranging from 64.4 ± 12.4 for those with 0–1 comorbidity to 71.6 ± 10.9 for those with ≥ 4 comorbidities (*p* < 0.001) (Table 2). This was also observed in non-CVD patients.

**Table 2:** Hospital healthcare utilisation in patients with AF and non-CVD disease by multimorbidity group

	AF				Non-CVD			
Number of comorbidities	0–1	2–3	> 4	*p*-value	0–1	2–3	> 4	*p*-value
*N*	1,354	2,187	1,586		134,561	73,654	13,649	
Age (SD)	64.4 ± 12.4	67.5 ± 12.4	71.6 ± 10.9	< 0.001	52.2 ± 17.9	54.5 ± 18.2	59.1 ± 17.1	< 0.001
Female, *n* (%)	603 (45)	976 (45)	695 (44)	0.875	74,173 (55)	41,951 (57)	8,024 (59)	< 0.001
Number of comorbidities, *n* (%)	1.0 ± 0.0	2.4 ± 0.5	5.2 ± 1.6	< 0.001	1.0 ± 0.2	2.3 ± 0.5	4.6 ± 1.0	< 0.001
Hypertension, *n* (%)	0	38 (2)	46 (3)	< 0.001	851 (1)	963 (1)	345 (3)	< 0.001
Heart Failure, *n* (%)	0	61 (3)	161 (10)	< 0.001	0	0	0	–
Diabetes, *n* (%)	0	35 (2)	83 (5)	< 0.001	868 (1)	1,964 (3)	871 (6)	< 0.001
Myocardial infarction, *n* (%)	0	15 (1)	23 (2)	< 0.001	0	0	0	–
Valvular disease, *n* (%)	0	2 (0.1)	7 (0.4)	0.008	0	0	0	–
Syncope, *n* (%)	0	12 (1)	14 (1)	0.003	173 (0.1)	251 (0.3)	108 (1)	< 0.001
Chronic kidney disease, *n* (%)	0	30 (1)	110 (7)	< 0.001	327 (0.2)	1,005 (1)	598 (4)	< 0.001
Cancer, *n* (%)	87 (6)	502 (23)	614 (39)	< 0.001	18,596 (28)	20,857 (28)	5,756 (42)	< 0.001
Prior cerebrovascular event*, *n* (%)	0	50 (2)	50 (3)	< 0.001	847 (1)	900 (1)	314 (2)	< 0.001
COPD, *n* (%)	0	59 (3)	124 (8)	< 0.001	1,150 (1)	1,295 (2)	581 (4)	< 0.001
*Hospital healthcare utilisation*								
Outpatient visits (SD)	1.8 ± 1.5	5.2 ± 3.5	12.6 ± 6.9	< 0.001	1.9 ± 2.0	5.1 ± 3.8	11.7 ± 6.8	< 0.001
Outpatient visits, median (Q1, Q3)	1 (1, 2)	4 (3, 6)	11 (8, 15)		1 (1, 2)	4 (3, 6)	10 (7, 14)	
ED Visits (SD)	0.3 ± 0.7	0.7 ± 1.1	1.7 ± 1.9	< 0.001	0.1 ± 0.3	0.3 ± 0.7	0.9 ± 1.5	< 0.001
ED visits, median (Q1, Q3)	0 (0, 0)	0 (0, 1)	1 (0, 3)		0 (0, 0)	0 (0, 0)	0 (0, 1)	
In hospital days (SD)	0.6 ± 2.3	3.0 ± 6.6	8.2 ± 12.0	< 0.001	0.8 ± 4.5	2.1 ± 7.0	5.7 ± 11.7	< 0.001
In hospital days, median (Q1, Q3)	0 (0, 0)	0 (0, 3)	3 (0, 11)		0 (0, 0)	0 (0, 0)	0 (0, 6)	
Medical specialties, mean (SD)	1.1 ± 0.4	2.4 ± 0.8	4.1 ± 0.9	< 0.001	1.1 ± 0.4	2.2 ± 0.7	4.0 ± 0.8	< 0.001
*Medical specialist*								
Anaesthesiology, *n* (%)	0	27 (1)	94 (6)	< 0.001	1,275 (1)	3,057 (4)	1,456 (11)	< 0.001
Cardiology, *n* (%)	1,158 (86)	1,853 (85)	1,416 (89)	< 0.001	5,268 (4)	6,530 (9)	2,638 (19)	< 0.001
Cardiothoracic surgery, *n* (%)	0	57 (3)	30 (2)	< 0.001	193 (0.1)	219 (0.3)	84 (0.6)	< 0.001
Clinical genetics, *n* (%)	0	26 (1)	15 (1)	< 0.001	7,724 (6)	3,746 (5)	590 (4)	< 0.001
Dermatology, *n* (%)	0	168 (8)	334 (21)	< 0.001	9,323 (7)	9,665 (13)	2,954 (22)	< 0.001
Gastroenterology, *n* (%)	18 (1)	183 (8)	305 (19)	< 0.001	4,154 (3)	6,488 (9)	2,676 (20)	< 0.001
General surgery, *n* (%)	4 (0.3)	241 (11)	551 (35)	< 0.001	7,531 (5)	13,889 (19)	5,305 (39)	< 0.001
Geriatrics, *n* (%)	0	70 (3)	128 (8)	< 0.001	945 (0.7)	1,445 (2)	660 (5)	< 0.001
Gynaecology, *n* (%)	0	119 (5)	102 (6)	< 0.001	12,976 (10)	11,701 (16)	2,366 (17)	< 0.001
Internal medicine, *n* (%)	43 (3)	573 (26)	861 (54)	< 0.001	18,026 (13)	21,792 (30)	7,356 (54)	< 0.001
Neurology, *n* (%)	41 (3)	322 (15)	468 (30)	< 0.001	95,575 (71)	13,278 (18)	5,001 (37)	< 0.001
Neurosurgery, *n* (%)	0	23 (1)	66 (4)	< 0.001	1,970 (2)	3,147 (4)	1,206 (9)	< 0.001
Ophthalmology, *n* (%)	0	150 (7)	312 (20)	< 0.001	12,391 (9)	8,844 (12)	3,141 (23)	< 0.001
Orthopaedic surgery, *n* (%)	0	114 (5)	318 (20)	< 0.001	6,536 (5)	7,874 (11)	3,302 (24)	< 0.001
Otorhinolaryngology, *n* (%)	0	119 (5)	263 (17)	< 0.001	8,085 (6)	8,018 (11)	2,771 (20)	< 0.001
Physiatry Rehabilitation, *n* (%)	0	17 (0.8)	24 (2)	< 0.001	233 (0.2)	2,108 (3)	624 (5)	< 0.001
Plastic surgery, *n* (%)	0	34 (2)	71 (5)	< 0.001	2,299 (2)	4,323 (6)	1,663 (12)	< 0.001
Psychiatry, *n* (%)	0	3 (0.1)	4 (0.3)	0.006	1,141 (0.9)	356 (0.5)	89 (1)	< 0.001
Pulmonology, *n* (%)	0	272 (12)	468 (30)	< 0.001	4,110 (3)	6,693 (9)	3,016 (22)	< 0.001
Radiotherapy, *n* (%)	87 (6)	175 (8)	201 (13)	< 0.001	3,841 (3)	6,723 (9)	2,494 (18)	< 0.001
Rheumatology, *n* (%)	3 (0.2)	119 (5)	206 (13)	< 0.001	2,982 (2)	6,115 (8)	2,061 (15)	< 0.001
Urology, *n* (%)	0	169 (8)	306 (19)	< 0.001	6,794 (5)	7,241 (10)	2,348 (17)	< 0.001

*AF* atrial fibrillation, *CVD* cardiovascular disease, *COPD* chronic obstructive pulmonary disease, *ED* emergency department

* Cerebrovascular event was defined as intracerebral hematoma, chronic subdural hematoma/hygroma, cerebrovascular accident/Transient ischemic attack, or, intracranial, subarachnoidal or intracerebral hemorrhage

There was a significant increase in healthcare utilisation based on the number of comorbidities (Table 2). The number of outpatient visits, emergency department visits, and in-hospitals days increased significantly in AF patients depending on the number of comorbidities. The number of outpatient visits, emergency department visits and in-hospital days were greater in AF patients with ≥ 4 comorbidities compared to non-CVD patients with ≥ 4 comorbidities, (all *p* < 0.001). The correlation for inpatient visits and the number of diagnosis was stronger in the AF group (*r* = 0.3393) compared to the non-CVD group (*r* = 0.2544), with a statistically significant difference (*z* = 6.0899, *p* < 0.0001). Similarly, the correlation for outpatient visits was stronger in the AF group (*r* = 0.6676) compared to the non-CVD group (*r* = 0.6410), with a statistically significant difference (*z* = 3.0408, *p* = 0.0024).

**Fig. 2:**
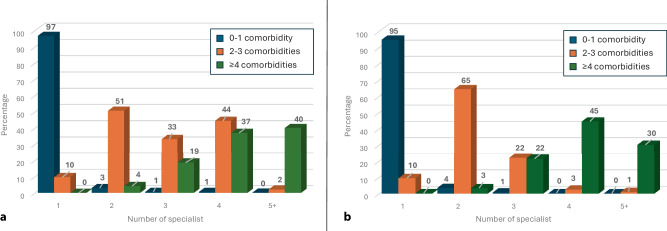
Bar graph demonstrating the number of specialists seen by patients with atrial fibrillation (**a**) and non-cardiovascular disease (**b**). Patients with AF and 2–3 comorbidities (shown in the orange bar) or > 4 comorbidities (shown in the green bar) saw 4 and 5+ specialist more frequently than patients with non-cardiovascular disease

The number of medical specialists also increased incrementally across multimorbidity categories up to a mean of 4.1 in AF patients with ≥ 4 comorbidities (*p* < 0.001) and was higher than in non-CVD patients (Fig. 2). The most frequently consulted specialists are shown in Table S1 (see Electronic Supplementary Material).

### Age in healthcare utilisation in AF versus non-CVD patients

Among AF patients, the mean number of comorbidities increased with age, ranging from 2.4 ± 1.6 for 18–59 years to 3.5 ± 2.0 for ≥ 75 years (*p* < 0.001) (Table S1 (see Electronic Supplementary Material)). In non-CVD patients, the mean number of comorbidities also increased with age, but this trend was less observed.

There was a significant age-related increase in healthcare utilisation based in both AF and non-CVD patients (Table S1, see Electronic Supplementary Material). The number of outpatient visits, emergency department visits, and in-hospital days among AF patients increased significantly depending on age and were higher across all age groups compared non-CVD patients (all *p* < 0.001). The most significant correlation was observed between age and the emergency department visits, and was modest in both groups but significantly different: *r* = 0.0795 in the AF group vs *r* = 0.1440 in the no-CVD group (*z* = −4.27, *p* = 0.00002).

The number of specialists involved in patient care also increased with age. Among patientsaged ≥ 75 years, 20% of patients with AF consulted ≥ 5 specialists, compared to 5% non-CVD patients (*p* < 0.001). The most frequently consulted specialists for AF and non-CVD across age groups are shown in Table S1 (see Electronic Supplementary Material).

### Association between comorbidities and healthcare utilisation

**Fig. 3:**
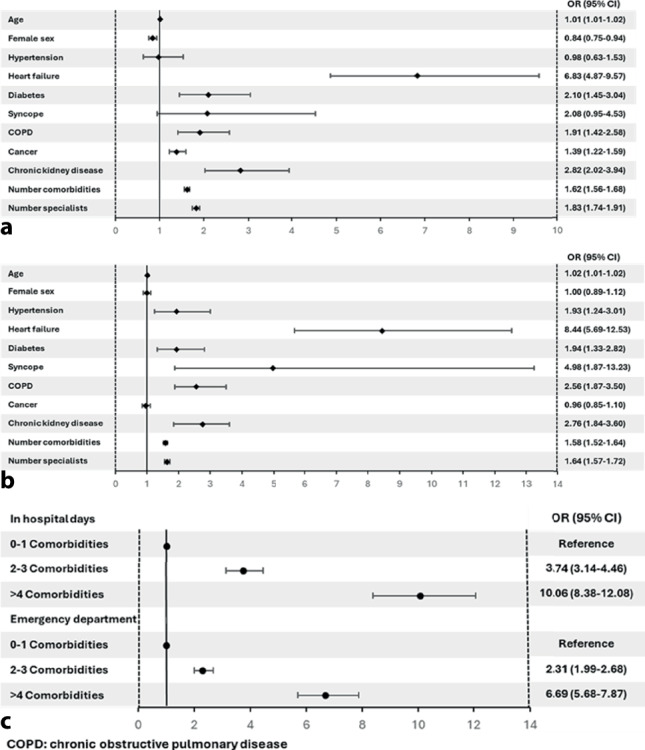
Logistic regression in patients with atrial fibrillation demonstrating risk of in hospital stay (**a**), emergency department presentation **b** for comorbidities and **c** for in hospital and emergency department visits in patients with atrial fibrillation for multimorbidity

In patients with AF, univariate analysis demonstrated age, number of comorbidities, number of specialists, heart failure, diabetes, COPD, cancer, and chronic kidney disease were all significantly associated with an increased risk of in-hospital stay (Fig. 3a), which remained following adjustment for age and sex. Age, number of comorbidities, number of specialists, hypertension, heart failure, diabetes, syncope, COPD, and chronic kidney disease were all significantly associated with an increased risk of emergency department visits (Fig. 3b) and these associations also remaind after adjustment for age and sex. Multimorbidity was strongly associated with an increased risk of in-hospital stay (≥ 4 comorbidities: OR 10.6, 95% CI: 8.4–12.1) and increased risk of emergency department visits (≥ 4 comorbidities: OR 6.7, 95% CI: 5.7–7.9) (Fig. 3c).

## Discussion

This study presents data of hospital healthcare utilisation in the Netherlands, demonstrating that AF patients present with more comorbidities than non-CVD patients. Compared to non-CVD patients, AF patients had significantly more outpatient visits, emergency department visits and a longer in-hospital stay. Additionally, patients with AF consulted more specialists. Multimorbidity played a significant role in both AF and non-CVD patients, though its impact was more pronounced in patients with AF, being associated with more outpatient visits and longer hospital stays. Finally, age was an important factor associated with hospital healthcare utilisation, also more pronounced in patients with AF.

### Hospital healthcare utilisation of patients with AF

In our study, patients with AF had significantly higher hospital healthcare utilisation than non-CVD patients, including twice as many outpatient visits and more than double the length of in-hospital days. Although the available data are limited, our findings are comparable to previous data [4, 11]. In patients with incident AF compared to a propensity-matched cohort, patients with AF had a significantly higher all-cause inpatient visits and cardiovascular-related emergency room visits [11]. The reasons for hospitalisations in patients with AF have been studied, showing that CVD, including AF itself, are predominantly the primary cause [4, 12–14].

Patients with AF are complex, and their care often involves multiple specialists. A study assessing the feasibility of aligning medical specialty appointments in patients with multimorbidity in the hospital outpatient setting [15], showed that it was possible, but remains challenging. Clearly, the development of multidisciplinary clinics and care managed by single case-holder or care coordinator may improve patient care and reduce healthcare costs.

### Role of multimorbidity in hospital healthcare utilisation in AF

Multimorbidity increases the burden on healthcare systems, leading to higher costs and polypharmacy [12]. In elderly patients (mean age 75 years), 64% had five or more comorbidities [16]. In our study, multimorbidity was associated with greater healthcare utilisation, including more outpatient visits, emergency department visits and longer in-hospital stays. Prior studies in non-AF cohorts have demonstrated the impact of multimorbidity on healthcare utilisation [17]. However, there is limited data on the impact of hospital healthcare utilisation specifically in relation to multimorbidity in AF patients.

Interestingly, we observed a high prevalence of non-cardiovascular comorbidities, particularly cancer which was present in 24% of patients with AF. The association between AF and cancer is known, however this is a complex relationship due to shared risk factors, but only limited data are available [18]. A recent study also found an association, albeit with lower percentages for both incidence and prevalence of cancer [19]. This association may clearly differ depending on the age and type of patients being studied. Nevertheless, in patients with AF, undiagnosed cancer should be recognised as one of the underlying comorbidities.

It is important to recognise the economic burden multimorbidity can pose on the healthcare system, highlighting the need to focus on ways of reducing this burden [20]. The importance of addressing comorbidities is currently studied in the European Heart Rhythm Associations (EHRA-PATHS) project, which aims to increase the diagnosis and treatment of comorbidities through creating new and effective care pathways in patients with AF [21].

### Role of age in healthcare utilisation in AF

Age is a well-established risk factor for AF [22]. Nevertheless, the incidence of AF is also increasing among young individuals with associated risk factors and comorbidities [22, 23]. In a study of patients with a mean age of AF onset at 46 years, 9 out of 10 had risk factors or comorbidities [23]. Data from a matched cohort study comparing those aged < 65 years to > 65 years, showed a similarly increased risk of all-cause inpatient, outpatient, and emergency room visits for both age groups for patients with AF compared to non-AF controls [11]. This is contrasts with data from the Nationwide Inpatients Sample database, which also compared those aged < 65 years to > 65 years and observed that older patients were more likely to have a longer in-hospital stay and, following discharge, were less likely to return home [24].

Hospital healthcare utilisation may also be influenced by differences in management strategies based on age. Younger patients more often undergo rhythm control strategies, such as cardioversion or ablation [25] This could explain the higher hospital healthcare utilisation of patients in our study, with this population requiring more interventions such as cardioversion or rhythm control management. We acknowledge that healthcare burden in patients with AF may be driven not only by AF itself but also by associated comorbidities. Our analysis reflects this real-world complexity. While we used calendar age and a non-CVD comparator group, biological age or frailty may better capture underlying drivers of burden, this is an important direction for future research.

### Strengths and limitations

The strengths of our study included the use of electronic health records from 226,991 patients, all of which have been extracted uniformly, indicating the methodology utilised could be replicated across hospitals in the Netherlands. This data collection method enabled the inclusion of typically underrepresented individuals in research allowing for more diversity in the population studied [26].

However, there are several limitations. First, some comorbidities are underrepresented such as hypertension. Only 2% of the AF patients reported having hypertension, which is contrary to existing knowledge, as hypertension is recognised as one of the most prevalent comorbidities [27]. Second, we were restricted of data, limiting our ability to perform longitudinal analysis. Third, this cohort was taken from hospital data therefore representing a potentially sicker population, as we did not have access to patients with AF in the general community for comparison. However, this cohort does highlight the complexity of AF patients who present frequently to our healthcare system. Fourth, to isolate the association of AF with healthcare use, we compared AF patients with comorbid CVD to a control group without AF or CVD, excluding those with CVD alone. While this approach strengthens internal validity, we acknowledge that excluding CVD-only patients may underestimate the broader burden of non-AF cardiovascular conditions. Additionally, as all patients with AF also had comorbid CVD, it was not possible to isolate the effect of AF alone on healthcare utilisation. While this reflects real-world comorbidity patterns, it limits our ability to distinguish whether observed differences in healthcare utilisation are specifically attributable to AF or to associated cardiovascular conditions. We acknowledge this limitation and suggest that future studies explore stratification within the AF population where feasible. Finally, we did not have access to complete patient-level data such as medication use, or pathology, or procedures undertaken.

## Conclusion

In this large cohort of patients in the Netherlands, patients with AF had significantly more hospital healthcare utilisation. Importantly, multimorbidity and age both played a significant role. With an increase in the number of comorbidities and increasing age, there was a significantly greater increase in hospital healthcare utilisation in both AF and non-CVD patients, but this increase was consistently higher in patients with AF. Our findings highlight the need for more cohesive care pathways for specialists managing patients with AF.

## Supplementary Information

Table S1—Hospital healthcare utilization in patients with AF and non-CVD disease by age group

## References

[1] Dong XJ, Wang BB, Hou FF, et al. Global burden of atrial fibrillation/atrial flutter and its attributable risk factors from 1990 to 2019. Europace. 2023;25:793–803. 36603845 10.1093/europace/euac237 PMC10062373

[2] Freeman JV, Wang Y, Akar J, Desai N, Krumholz H. National trends in atrial fibrillation hospitalization, readmission, and mortality for medicare beneficiaries, 1999–2013. Circulation. 2017;135:1227–39. 28148599 10.1161/CIRCULATIONAHA.116.022388

[3] Gallagher C, Hendriks JM, Giles L, et al. Increasing trends in hospitalisations due to atrial fibrillation in Australia from 1993 to 2013. Heart. 2019;105:1358–63. 30936408 10.1136/heartjnl-2018-314471

[4] Dong Z, Du X, Lu S, et al. Incidence and predictors of hospitalization in patients with atrial fibrillation: results from the Chinese atrial fibrillation registry study. BMC Cardiovasc Disord. 2021;21:146. 33740910 10.1186/s12872-021-01951-5 PMC7980549

[5] Van Deutekom C, Geelhoed B, Van Munster BC, et al. Cardiovascular and renal multimorbidity increase risk of atrial fibrillation in the PREVEND cohort. Open Heart. 2023;10(2):e002315. 10.1136/openhrt-2023-002315. 10.1136/openhrt-2023-002315 PMC10357795 37460268

[6] Nguyen BO, Weberndorfer V, Crijns HJ, et al. Prevalence and determinants of atrial fibrillation progression in paroxysmal atrial fibrillation. Heart. 2022;109:186–94. 35858774 10.1136/heartjnl-2022-321027 PMC9872250

[7] WHO. World health organization: Multimorbitity. https://iris.who.int/bitstream/handle/10665/252275/9789241511650-eng.pdf?sequence=1. Accessed 1 May 2024.

[8] Jani BD, Nicholl BI, McQueenie R, et al. Multimorbidity and co-morbidity in atrial fibrillation and effects on survival: findings from UK Biobank cohort. Europace. 2018;20(FI_3):f329–f36. 29112751 10.1093/europace/eux322 PMC6277149

[9] Folmer K, Mot E. Diagnosis and treatment combinations in Dutch hospitals 2003. 2003. https://www.researchgate.net/publication/242185877_Diagnosis_and_treatment_combinations_in_Dutch_hospitals#fullTextFileContent. Accessed 18 Mar 2024.

[10] Healthcare Cost and Utilization Project (HCUP). Beta clinical classifications software (CCS) for ICD-10-CM/PCS. 2018. https://hcup-us.ahrq.gov/toolssoftware/ccs10/ccs10.jsp. Accessed 18 Mar 2024.

[11] Deshmukh A, Iglesias M, Khanna R, Beaulieu T. Healthcare utilization and costs associated with a diagnosis of incident atrial fibrillation. Heart Rhythm O2. 2022;3:577–86. 36340482 10.1016/j.hroo.2022.07.010 PMC9626881

[12] Chamberlain AM, Alonso A, Gersh BJ, et al. Multimorbidity and the risk of hospitalization and death in atrial fibrillation: a population-based study. Am Heart J. 2017;185:74–84. 28267478 10.1016/j.ahj.2016.11.008 PMC5343767

[13] van Doorn S, Tavenier A, Rutten FH, et al. Risk of cardiac and non-cardiac adverse events in community-dwelling older patients with atrial fibrillation: a prospective cohort study in the Netherlands. BMJ Open. 2018;8:e21681. 30139900 10.1136/bmjopen-2018-021681 PMC6112390

[14] Frausing MH, Van De Lande ME, Linz D, et al. Healthcare Utilisation and quality of life according to atrial fibrillation burden, episode frequency and duration. Heart. 2024;110(16):1030–1039. 10.1136/heartjnl-2024-324016. 10.1136/heartjnl-2024-324016 PMC11287643 38944418

[15] Bell C, Appel CW, Frolich A, Prior A, Vedsted P. Improving health care for patients with Multimorbidity: a mixed-methods study to explore the feasibility and process of aligning scheduled outpatient appointments through collaboration between medical specialties. Int J Integr Care. 2022;22:17. 35340347 10.5334/ijic.6013 PMC8896239

[16] Abu HO, Saczynski J, Mehawej J, et al. Multimorbidity, physical frailty, and self-rated health in older patients with atrial fibrillation. BMC Geriatr. 2020;20:343. 32917137 10.1186/s12877-020-01755-w PMC7488548

[17] Rodrigues LP, de Oliveira Rezende AT, Delpino FM, et al. Association between multimorbidity and hospitalization in older adults: systematic review and meta-analysis. Age Ageing. 2022;51(7):afac155. 10.1093/ageing/afac155. 10.1093/ageing/afac155 PMC9308991 35871422

[18] Farmakis D, Parissis J, Filippatos G. Insights into onco-cardiology: atrial fibrillation in cancer. J Am Coll Cardiol. 2014;63:945–53. 24361314 10.1016/j.jacc.2013.11.026

[19] Chen Q, van Rein N, van der Hulle T, et al. Coexisting atrial fibrillation and cancer: time trends and associations with mortality in a nationwide Dutch study. Eur Heart J. 2024;45(25):2201–2213. 10.1093/eurheartj/ehae222. 10.1093/eurheartj/ehae222 PMC11231645 38619538

[20] Hindricks G, Potpara T, Dagres N, et al. 2020 ESC Guidelines for the diagnosis and management of atrial fibrillation developed in collaboration with the European Association for Cardio-Thoracic Surgery (EACTS): The Task Force for the diagnosis and management of atrial fibrillation of the European Society of Cardiology (ESC) Developed with the special contribution of the European Heart Rhythm Association (EHRA) of the ESC. Eur Heart J. 2021;42:373–498. 32860505 10.1093/eurheartj/ehaa612

[21] Heidbuchel H, Van Gelder IC, Desteghe L. ESC and EHRA lead a path towards integrated care for multimorbid atrial fibrillation patients: the horizon 2020 EHRA-PATHS project. Eur Heart J. 2021;43(15):1450–1452. 10.1093/eurheartj/ehab672 10.1093/eurheartj/ehab672 34694355

[22] Morseth B, Geelhoed B, Linneberg A, et al. Age-specific atrial fibrillation incidence, attributable risk factors and risk of stroke and mortality: results from the MORGAM Consortium. Open Heart. 2021;8(2):e001624. 10.1136/openhrt-2021-001624. 10.1136/openhrt-2021-001624 PMC8330568 34341095

[23] De With RR, Marcos EG, Van Gelder IC, Rienstra M. Atrial fibrillation progression and outcome in patients with young-onset atrial fibrillation. Europace. 2018;20:1750–7. 29518195 10.1093/europace/euy028

[24] Naderi S, Wang Y, Miller AL, et al. The impact of age on the epidemiology of atrial fibrillation hospitalizations. Am J Med. 2014;127(158):e1–e7. 10.1016/j.amjmed.2013.10.005 PMC4436031 24332722

[25] Kirchhof P, Camm AJ, Goette A, et al. Early Rhythm-Control Therapy in Patients with Atrial Fibrillation. N Engl J Med. 2020;383:1305–16. 32865375 10.1056/NEJMoa2019422

[26] Witham MD, Cooper R, Missier P, et al. Researching multimorbidity in hospital: can we deliver on the promise of health informatics? Eur Geriatr Med. 2023;14:765–8. 37227692 10.1007/s41999-023-00753-6 PMC10447588

[27] Verdecchia P, Angeli F, Reboldi G. Hypertension and Atrial Fibrillation: Doubts and Certainties From Basic and Clinical Studies. Circ Res. 2018;122:352–68. 29348255 10.1161/CIRCRESAHA.117.311402

