# Supplementary material for: Hospital healthcare utilisation in patients with atrial fibrillation: the role of multimorbidity and age
Source: Neth Heart J. 2025 Jul 18;33(9):270–80. doi: 10.1007/s12471-025-01968-x (PMC12364794; doi:10.1007/s12471-025-01968-x)
Supplement: Supplementary file 1 — Table S1—Hospital healthcare utilization in patients with AF and non-CVD disease by age group [file 12471_2025_1968_MOESM1_ESM.docx]

**Table S1 –** **Hospital healthcare utilization in patients with AF and non-CVD disease by age group**

|  |  | **AF** |  |  |  |  | **Non-CVD** |  |  |
| --- | --- | --- | --- | --- | --- | --- | --- | --- | --- |
|  | **18-59** | **60-74** | **>75** | **p-value** |  | **18-59** | **60-74** | **>75** | **p-value** |
|  | **1107** | **2407** | **1613** |  |  | **128961** | **66566** | **26337** |  |
| Age (SD) | 49.9±8.9 | 67.8±4.1 | 80.5±4.5 | <0.001 |  | 40.9±12.2 | 66.8±4.2 | 80.4±4.6 | <0.001 |
| Female, n (%) | 473 (43) | 981 (41) | 820 (51) | <0.001 |  | 77915 (60) | 32734 (49) | 13499 (51) | <0.001 |
| Nunmber comorbidities, mean (SD) | 2.4±1.6 | 2.8±1.8 | 3.5±2.0 | <0.001 |  | 1.5±1.0 | 1.7±1.1 | 1.8±1.3 | <0.001 |
| Hypertension, n (%) | 13 (1) | 36 (2) | 35 (2) | 0.1 |  | 939 (0.7) | 890 (1) | 330 (1) | <0.001 |
| Heart Failure, n (%) | 18 (2) | 81 (3) | 123 (8) | <0.001 |  | 0 | 0 | 0 | - |
| Diabetes, n (%) | 21 (2) | 51 (2) | 46 (3) | 0.189 |  | 2063 (1.6) | 1257 (2) | 383 (2) | <0.001 |
| Myocardial infarction, n (%) | 3 (0.3) | 16 (0.7) | 19 (1) | 0.021 |  | 0 | 0 | 0 | - |
| Valvular disease, n (%) | 10 (0.9) | 20 (0.8) | 33 (2) | 0.002 |  | 0 | 0 | 0 | - |
| Syncope, n (%) | 5 (0.5) | 12 (0.5) | 9 (0.6) | 0.926 |  | 281 (0.2) | 142 (0.2) | 109 (0.4) | <0.001 |
| Chronic kidney disease, n (%) | 8 (0.7) | 67 (3) | 79 (5) | <0.001 |  | 654 (0.5) | 882 (1) | 555 (2) | <0.001 |
| Cancer, n (%) | 187 (17) | 538 (22) | 478 (30) | <0.001 |  | 16446 (13) | 20112 (30) | 8651 (33) | <0.001 |
| Cerebrovascular event*, n (%) | 14 (1) | 44 (1.8) | 42 (3) | 0.039 |  | 750 (0.6) | 719 (1.1) | 592 (2.3) | <0.001 |
| COPD, n (%) | 14 (1) | 92 (4) | 77 (5) | <0.001 |  | 858 (0.7) | 1556 (2) | 612 (2) | <0.001 |
|  |  |  |  |  |  |  |  |  |  |
| Outpatient visits (SD) | 5.4±5.9 | 6.4±6.1 | 7.8±7.5 | <0.001 |  | 3.3±3.9 | 3.9±4.4 | 4.1±4.2 | <0.001 |
| Outpatient visits, median (Q1, Q3) | 3 (2,7) | 4 (2,9) | 6 (3,10) |  |  | 2 (1,4) | 2 (1,5) | 3 (1,5) |  |
| ED Visits (SD) | 0.8±1.3 | 0.9±1.4 | 1.1±1.5 | <0.001 |  | 0.2±0.6 | 0.2±0.6 | 0.3±0.8 | <0.001 |
| ED visits, median (Q1, Q3) | 0 (0,1) | 0 (0,1) | 1 (0,2) |  |  | 0 (0,0) | 0 (0,0) | 0 (0,0) |  |
| In hospital days (SD) | 3.0±7.6 | 3.5±8.2 | 5.3±9.3 | <0.001 |  | 1.3±6.0 | 1.8±6.5 | 2.3±6.2 | 0.3801 |
| In hospital days, median (Q1, Q3) | 0 (0,2) | 0 (0,3) | 0 (0,7) |  |  | 0 (0,0) | 0 (0,0) | 0 (0,0) |  |
| **Involved medical specialties, n (%)** |  |  |  |  |  |  |  |  |  |
| 1 | 645 (58) | 767 (32) | 275 (17) |  |  | 83629 (65) | 38479 (58) | 12862 (49) |  |
| 2 | 308 (28) | 572 (24) | 334 (21) |  |  | 29964 (23) | 16027 (24) | 6878 (26) |  |
| 3 | 171 (15) | 483 (20) | 378 (23) | <0.001 |  | 9810 (8) | 6958 (11) | 3620 (14) | <0.001 |
| 4 | 84 (8) | 303 (13) | 305 (19) |  |  | 3593 (3) | 3038 (5) | 1768 (7) |  |
| 5+ | 79 (7) | 282 (13) | 321 (20) |  |  | 1965 (2) | 2064 (3) | 1209 (5) |  |
| Involved specialties, mean (SD) | 2.1±1.2 | 2.5±1.4 | 3.0±1.4 | <0.001 |  | 1.5±0.9 | 1.7±1.0 | 1.9±1.1 | <0.001 |
| **Medical Specialist** |  |  |  |  |  |  |  |  |  |
| Anaesthesiology, n (%) | 22 (2) | 47 (2) | 52 (3) | 0.022 |  | 3047 (2) | 1939 (3) | 802 (3) | <0.001 |
| Cardiology, n (%) | 866 (78) | 2136 (89) | 1425 (88) | <0.001 |  | 6984 (5) | 5073 (8) | 2469 (9) | <0.001 |
| Cardiothoracic surgery, n (%) | 38 (3) | 40 (2) | 9 (0.6) | <0.001 |  | 298 (0.2) | 173 (0.3) | 25 (0.1) | <0.001 |
| Clinical genetics, n (%) | 21 (2) | 18 (0.8) | 2 (0.1) | <0.001 |  | 9614 (8) | 2142 (3 | 304 (1) | <0.001 |
| Dermatology, n (%) | 55 (5) | 210 (9) | 237 (15) | <0.001 |  | 11517 (9) | 6774 (10) | 3651 (14) | <0.001 |
| Gastroenterology, n (%) | 109 (10) | 212 (9) | 185 (12) | 0.021 |  | 7299 (6) | 4357 (7) | 1662 (6) | <0.001 |
| General surgery, n (%) | 114 (10) | 360 (15) | 322 (20) | <0.001 |  | 14963 (12) | 8116 (12) | 3646 (14) | <0.001 |
| Geriatrics, n (%) | 6 (0.5) | 45 (2) | 147 (9) | <0.001 |  | 94 (0.1) | 826 (1) | 2130 (8) | <0.001 |
| Gynaecology, n (%) | 77 (7) | 66 (3) | 78 (5) | <0.001 |  | 23163 (18) | 2783 (4) | 1097 (4) | <0.001 |
| Internal medicine, n (%) | 278 (25) | 646 (27) | 553 (34) | <0.001 |  | 27425 (21) | 14989 (23) | 4760 (18) | <0.001 |
| Neurology, n (%) | 143 (13) | 374 (16) | 314 (20) | <0.001 |  | 15097 (12) | 8734 (13) | 4023 (15) | <0.001 |
| Neurosurgery, n (%) | 13 (1) | 43 (2) | 33 (2) | 0.224 |  | 3320 (3) | 2243 (3) | 760 (3) | <0.001 |
| Ophthalmology, n (%) | 57 (5) | 197 (8) | 208 (13) | <0.001 |  | 10119 (8) | 9330 (14) | 4927 (191) | <0.001 |
| Orthopaedic surgery, n (%) | 60 (5) | 177 (7) | 195 (12) | <0.001 |  | 10093 (8) | 5106 (8) | 2513 (10) | <0.001 |
| Otorhinolaryngology, n (%) | 48 (4) | 172 (7) | 162 (10) | <0.001 |  | 9883 (8) | 6295 (10) | 2696 (10) | <0.001 |
| Physiatry Rehabilitation, n (%) | 9 (0.8) | 25 (1) | 7 (0.4) | 0.944 |  | 2084 (2) | 724 (1) | 157 (0.6) | <0.001 |
| Plastic surgery, n (%) | 24 (2) | 48 (2) | 33 (2) | 0.846 |  | 5454 (4) | 2221 (3) | 610 (2) | <0.001 |
| Psychiatry, n (%) | 2 (0.1) | 4 (0.2) | 1 (0.1) | <0.001 |  | 1395 (1) | 159 (0.2) | 32 (0.1) | <0.001 |
| Pulmonology, n (%) | 97 (9) | 371 (15) | 272 (17) | <0.001 |  | 5914 (5) | 5733 (9) | 2172 (8) | <0.001 |
| Radiotherapy, n (%) | 108 (10) | 213 (9) | 142 (9) | 0.635 |  | 4303 (3) | 6120 (9) | 2635 (10) | <0.001 |
| Rheumatology, n (%) | 55 (5) | 152 (6) | 121 (8) | 0.029 |  | 6057 (5) | 3783 (6) | 1328 (5) | <0.001 |
| Urology, n (%) | 41 (4) | 229 (10) | 205 (13) | <0.001 |  | 6393 (5) | 7065 (16) | 2925 (11) | <0.001 |
| AF: atrial fibrillation; CVD: cardiovascular disease; COPD: chronic obstructive pulmonary disease; ED: emergency department  * Cerebrovascular event was defined as intracerebral hematoma, chronic subdural hematoma/hygroma, cerebrovascular accident/Transient ischemic attack, or, intracranial, subarachnoidal or intracerebral hemorrhage | | | | | | | | | |
